# Advanced Activity-Based Protein Profiling Application Strategies for Drug Development

**DOI:** 10.3389/fphar.2018.00353

**Published:** 2018-04-09

**Authors:** Shan Wang, Yu Tian, Min Wang, Min Wang, Gui-bo Sun, Xiao-bo Sun

**Affiliations:** ^1^Beijing Key Laboratory of Innovative Drug Discovery of Traditional Chinese Medicine (Natural Medicine) and Translational Medicine, Institute of Medicinal Plant Development, Chinese Academy of Medical Sciences and Peking Union Medical College, Beijing, China; ^2^Life and Environmental Science Research Center, Harbin University of Commerce, Harbin, China

**Keywords:** ABPP, isoTOP-ABPP, fluoPol-ABPP, qNIRF-ABPP, drug targets

## Abstract

Drug targets and modes of action remain two of the biggest challenges in drug development. To address these problems, chemical proteomic approaches have been introduced to profile targets in complex proteomes. Activity-based protein profiling (ABPP) is one of a growing number chemical proteomic approaches that uses small-molecule chemical probes to understand the interaction mechanisms between compounds and targets. ABPP can be used to identify the protein targets of small molecules and even the active sites of target proteins. This review focuses on the overall workflow of the ABPP technology and on additional advanced strategies for target identification and/or drug discovery. Herein, we mainly describe the design strategies for small-molecule probes and discuss the ways in which these probes can be used to identify targets and even validate the interactions of small molecules with targets. In addition, we discuss some basic strategies that have been developed to date, such as click chemistry-ABPP, competitive strategies and, recently, more advanced strategies, including isoTOP-ABPP, fluoPol-ABPP, and qNIRF-ABPP. The isoTOP-ABPP strategy has been coupled with quantitative proteomics to identify the active sites of proteins and explore whole proteomes with specific amino acid profiling. FluoPol-ABPP combined with HTS can be used to discover new compounds for some substrate-free enzymes. The qNIRF-ABPP strategy has a number of applications for *in vivo* imaging. In this review, we will further discuss the applications of these advanced strategies.

## Introduction

Two major challenges in the field of drug discovery are drug development and target identification (Schenone et al., 2013). The identification of drug targets, which is important for elucidating the mode of action, is of great significance in the process of drug discovery. Two drug discovery strategies are currently used: phenotype-based drug discovery and target-based drug discovery (Samsdodd, 2005). Phenotype-based drug discovery refers to the screening of small molecules or polypeptides in cells, tissues, or organs based on existing pharmacology. Target-based drug discovery involves first determining the targets and then identifying active molecules. With the rapid development of molecular biology, target-based drug discovery paradigm replaced the traditional phenotype-based approach, because it allowed an increased screening capacity and the definition of rational drug discovery programs. However, analysis of the process of target-based drug discovery showed that this screening platform did not effectively improve the productivity of pharmaceutical industry, but the time and cost increased significantly (Samsdodd, 2005). Due to the complexity of biological systems, phenotype-based strategies can provide more comprehensive evaluation of potential drugs and play an important role in drug development. In recent years, phenotype-based strategies have received increasing attention and have become the main method for drug discovery. These screening strategies are more efficient, effective and economical than other screening platforms.

Numerous technologies for identifying targets have recently been developed. Experimental approaches such as genomic and proteomic techniques are the primary tools for target identification. To complement experimental methods, a series of computational (*in silico*) tools have also been developed for target identification over the past two decades (Krysiak and Breinbauer, 2012; Yue et al., 2012). With the advancement of molecular biology and the advent of the post-genomic era, these technologies provide a solid technical basis for improving the efficiency of drug discovery; however, there remain many barriers for the identification of drug targets, and we need to overcome these barriers.

Activity-based protein profiling is a technology to identify the binding of small molecule probes with proteins and confirm direct interaction. It combines activity-based probe and proteomics technologies together to help us to understand the mechanisms of compounds and the modes of action (Kozarich, 2003; Cravatt et al., 2008). The ABPP-like experiments were firstly reported in the early 1970s to explore the mechanisms of penicillin (Blumberg and Strominger, 1972; Suginaka et al., 1972).

However, the term proteome was firstly proposed at a scientific conference in Italy in 1994 (Wilkins et al., 1996; Huber, 2003). The development of proteomics allows the use of ABPP in many areas, from studying enzyme classes, including proteases, kinases, phosphatases, glycosidases, and oxidoreductases, to studying uncharacterized enzymes. ABPP has contributed to our understanding of enzyme activity in specific physiological and pathological processes on a proteome-wide scale (Heal et al., 2011; Li et al., 2012). This review will discuss all aspects of the ABPP workflow in greater detail. Appropriate strategies are also very important before beginning ABPP-associated experiments. With the development of this field, an increasing number of advanced strategies have been applied in more areas, and we will discuss these strategies in a later section of this review.

## Abpp Workflow

**FIGURE 1:**
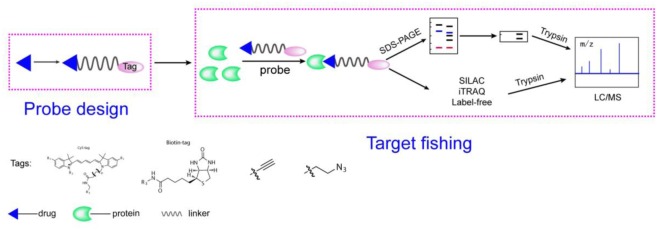
A general representation of the ABPP workflow. The probe is designed based on the structure of the compounds, is added to the proteome and binds to its target protein. Gel-based method or quantitative approaches (label-free, iTRAQ, SILAC) for chemical proteomics experiments.

Activity-based protein profiling workflow (**Figure 1**) will be discussed in the section and some important issues will be considered. Small-molecule probes are firstly designed and synthesized before ABPP progress begin, the basic chemical structure of a small-molecule probe consists of three parts: 1, a reactive group; 2, a linker site; and 3, a reporter group (Niphakis and Cravatt, 2014). In principle, the active group of small molecule interacts directly with the target protein and the reporter group to facilitate target fishing. Commonly used reporter groups are fluorescent groups, biotin, alkynes or azide, which can be modified by click chemistry methods to visualize protein targets. Depending on the selected reporting groups, different subsequent experiments can be carried out. For example, fluorescent groups can be used for rapid gel screening and the identification of the localization of small molecules in cells or animals, and biotin can be used for protein enrichment and then detected by mass spectrometry to identify target proteins.

After the probe is obtained, it is firstly subjected to rapid determination of working concentration and reaction time by using SDS-PAGE (Wright and Sieber, 2016). Typical workflows are as follows: (i) incubation of the probe with proteins, live cells, tissues, or animals to react with the target, (ii) for cc-probes, performing CuAAC to catalytically label the protein with a fluorescent group or other detectable labels followed by protein enrichment and pull-down assays, (iii) performing gel electrophoresis and fluorescence scanning or Western blotting (for detection of biotin) or quantitative proteomics to identify the target, and (iv) verifying the targets.

During the course of an ABPP project, there are many conditions that must be carefully considered. First, the probe can be incubated in cell lysates or in tissue homogenates *in vitro*. In this case, the conditions of the lysate are very important because the protein function and folding state must be retained to allow the protein to specifically bind to the probe molecule; Tris buffer or PBS are usually suitable (Speers and Cravatt, 2009). *In situ* labeling of cells in culture or *in vivo* labeling of mice via i.p. injection using an ABPP probe can be used to avoid this problem because in these conditions the probe interacts with the protein in a natural state. The caveat of the *in situ* method is that the probe-labeled protein may be metabolized. Some cytotoxic probes may also reduce the amount of protein recovered by killing the cells. However, these problems can be avoided by shortening the time of probing. Second, the selection of reporters should be considered. Biotin labeling can be used for protein enrichment, target identification, and Western blot verification. However, it has been reported that endogenous biotinylated proteins can enhance the noise signal and cause interference. Fluorescence detection is faster and cleaner than blot-based biotin detection and has no additional endogenous biotinylated protein signals (Charron et al., 2009). Other alternative approaches are emerging, such as IAF (immunoaffinity fluorescent) labeling (Yu et al., 2010), or the direct click-on-resin approach, to avoid the use of biotin (Cassiano et al., 2014). Finally, it is very important to comprehensively identify the potential target, including direct identification by pull-down Western blots and recombinant-protein interaction assays with small molecules. The next step is to confirm the mode of action between the proteins and compounds and to uncover the mechanisms by using SPR, ITC, and FP (fluorescence polarization immunoassay). Several assays of biological function are needed to test the associated pharmacological effects of the compounds.

### The Design of the Probe

A typical ABPP probe contains three groups: a reactive group, a linking group or binding group, and a reporter tag. For probe design, the first factor to consider is the reactivity of compound. Most probes are based on bioactive small molecules. So far, many ABPP probes have utilized electrophilic reactive groups, including epoxides, Michael-addition units, disulfides, lactones, β-lactams, and quinone compounds. These groups can react with serine, tyrosine, or glutamine to modulate enzyme activity (Bottcher and Sieber, 2008). However, there are many compounds that react with targets via non-covalent interactions. To overcome this problem, a more intuitive and unbiased strategy for identifying binding partners of unreactive NPs is to use photoaffinity labeling (PAL). PAL makes use of photoreactive moieties that are inert under standard synthetic-chemical and biological conditions but can be activated by UV light, generating highly reactive, transient species. Benzophenone, aliphatic and aromatic diazirines are the most commonly used PAL groups.

In the process of probe design, the choice of linking groups can also be critical. Linking groups can attach the reactive groups with the label groups together and reduce the impact of the label group on the reactive group. The choice of linker group is also significant for reducing non-specificity. In this basic form, a linker can take the form of an extended alkyl or polyethylene glycol (PEG) spacer. Furthermore, of late, the design of cleavable linkers for protein enrichment has received much attention, especially for the isoTOP-ABPP strategy; more details can be found in some other reviews (Leriche et al., 2012; Rudolf et al., 2013).

The other critical challenge in the process of probe design is the reporter group. The widely used reporters are the biotin-streptavidin system for pull-down assays and fluorescent reporters for imaging-based detection. Because of the existence of intrinsically biotinylated proteins, some non-specific background can interfere with the identification of targets; however, fluorescent reporters can be used to avoid this problem. An increasing number of studies are combining these two reporters to identify targets (Liao et al., 2017; Nasheri et al., 2013).

### Fishing the Targets

Fishing for targets by using probes is a very critical step, and different platforms have been developed. In this section, we will talk about two commonly used methods: gel-based and gel-free platforms.

#### Gel-Based Platform for ABPP

To investigate the targets of ABPs, the typical method is to utilize gel electrophoresis to separate proteins by one-dimensional (1D) or two-dimensional (2D) polyacrylamide gel electrophoresis (PAGE) and detect the proteins by Coomassie brilliant blue staining or silver staining to obtain specific bands. The bands are then cut, and LC/MS is used for protein identification. This is the original method for target identification; however, this method can introduce contaminants in the form of other proteins, especially keratin, which makes data analysis more challenging. Non-specific labeling of various proteins, especially of abundant and sticky proteins, in addition to that of the actual target proteins has been a major problem in ABPP. To address this limitation, Seung Park’s group have developed a new method called fluorescence difference in two-dimensional gel electrophoresis (FITGE) and employed it in the target identification of the anti-neuroinflammatory agent inflachromene (ICM) (Park et al., 2012; Lee et al., 2014). The platform can simultaneously label two or more different samples, such as control and treatment groups, with different fluorescent labels and then simultaneously perform two-dimensional gel electrophoresis. If one spot was labeled with two fluorescent labels, the labeling can be thought of as being non-specific, and only signals in the treatment group were identified by LC/MS. High-resolution gel electrophoresis can exclude some non-specific targets; however, 2D-PAGE always requires a large amount of protein, which can be difficult to obtain for some precious samples, especially human disease samples.

#### Gel-Free Approaches

Given the promiscuity of many small molecules and the complexity of the cellular proteome, a high-flux and high-accuracy method is necessary. With the development of mass spectrometers, ABPs coupled with quantitative chemical proteomics has been used to identify drug targets, which can achieve a high-throughput work platform while improving the accuracy of target-protein identification. Quantitative chemical proteomic approaches have been developed, including metabolic labeling (SILAC), chemical labeling (iTRAQ), and the label-free approach (Chen et al., 2017).

SILAC (stable isotope labeling by amino acids in culture) is a stable-isotope-based labeling method, which mainly involves elements of metabolic incorporation. iTRAQ, isobaric tags for relative and absolute quantification, which stands for isobaric tags for relative and absolute quantitation, uses chemical tagging to label different sample populations. These approaches need tags for quantification and identification. These tags result in mass differences that can be detected via MS and enable quantitation and comparison between multiple samples. Some researchers have used ABPP-SILAC and ABPP-iTRAQ to validate some examples. In 2014, Cravatt’s group examined the application of ABPP-SILAC to study the protein targets of the kinase inhibitor class of drugs, which includes the Bruton’s tyrosine kinase (BTK) inhibitor ibrutinib. A total of 29 probe targets were identified, including epidermal growth factor receptor and BTK (Lanning et al., 2014). Lin’s group explored the application of ABPP-iTRAQ to accurately identify the targets and mechanism of action of curcumin, a natural product with anti-inflammatory and anti-cancer properties. In total, 197 proteins were confidently identified from the HCT116 colon cancer cell line as binding targets of curcumin. Ingenuity pathway analysis (IPA) suggested that curcumin may exert its anticancer effects on multiple critical biological pathways, including the EIF2, eIF4/p70S6K, and mTOR signaling and mitochondrial dysfunction pathways (Wang et al., 2016). In iTRAQ-based mass spectrometry the protein is degraded into peptides and labeled at the final step of the entire process; therefore, in the event of an operational error, this process is irreversible. The ability of the ABPP-SILAC approach to identify a wide range of targets in an unbiased manner has been proved, especially for the identification of non-kinase off-target proteins. SILAC is limited by labeling efficiency. SILAC requires cell labeling, and cells often need to grow for at least 3 generations for high labeling efficiency, which is not suitable for some primary cells and tissues.

The label-free approach is another quantitative proteomic approach, which is generally cost-efficient and widely applicable compared to SILAC and iTRAQ. However, it was the need for very high reproducibility to allow run-run comparisons in label-free strategy. Artemisinin is the most potent of the anti-malarial drugs; however, the mechanism of action of artemisinin is not completely understood. Lin’s group used an unbiased chemical proteomic analysis to directly explore this mechanism in Plasmodium falciparum. This group designed and synthesized an alkyne-tagged artemisinin probe, combining click chemistry and the label-free method to identify 124 covalently binding protein targets of artemisinin, many of which are involved in essential biological processes of the parasite (Wang et al., 2015).

After the ABPP workflow is finished, the other important issue is to validate the targets. Once potential targets have been identified by ABPP, it is challenging to validate these targets and to verify their modes of action. Many approaches can be taken to assay the interactions between small molecules and targets; some of the commonly used approaches are as follows: (1) if the antibody is available or can be produced, the protein of interest may be enriched and then verified by Western blotting; (2) recombinant proteins can be used to perform the ABPP workflow and verify the interaction; (3) some biophysical methods, such as ITC (isothermal titration calorimetry), FPIA (fluorescence polarization immunoassay), SPR (surface plasmon resonance), and CTSA (cellular thermal shift assay), are should be used (Molina et al., 2013). (4) structural biology can also provide supportive evidence; (5) binding sites can be identified by LC-MS to further validate the direct site of interaction of proteins and small molecules, and if an amino acid modification can be identified, such as Cys or Ser, site-directed mutagenesis can be applied to identify these; and (6) the mode of action of small molecules can be very challenging, and it is necessary to apply many different biological and chemical tools, such as genetic methods and imaging technologies.

## Abpp Strategies

In recent years, ABPP technology has developed rapidly. To enhance the specificity and accuracy of this technology, some basic strategies, such as CC-ABPP (click chemistry-ABPP) and competitive-ABPP strategies, have been utilized in most studies. To expand the application of ABPP, some more advanced strategies have been developed, such as isoTOP-ABPP, fluoPol-ABPP and qNIRF-ABPP. These advanced strategies have different characteristics and are used in many areas from active sites identification to new potential compounds discovery and live imaging. The isoTOP-ABPP strategy can be used to directly identify active sites of target proteins; fluoPol-ABPP was used for the discovery of new small molecules based on specific enzymes; and qNIRF-ABPP provides us the opportunity to image the distribution of compounds and promote the development of preclinical diagnosis. We will discuss each strategy in greater detail.

### Basic Strategies

#### CC-ABPP (Click Chemistry-ABPP)

With the development of click chemistry, this method has been introduced into the field of ABPP technology. This method can overcome the limitations of bulky groups and enhance the cell permeability of the probes. By adding smaller alkyne or azide groups to the system, a single probe can be diversified with a variety of reporter groups without the need to develop new synthetic routes. The most widely used click chemistry reaction is the copper (I)-catalyzed azide-alkyne cycloaddition (CuAAC) between an azide and a terminal alkyne to generate a 1,4-disubstituted 1,2,3-triazole (Presolski et al., 2011; Martell and Weerapana, 2014). Concerns about the use of a cytotoxic copper species to catalyze the reaction promoted the development of a copper-free variant of this reaction, which utilizes a strained alkyne to accelerate the reaction (Chang et al., 2010).

To date, the use of CuAAC in living systems has been hindered by the toxicity of copper(I). Considerable cell death occurs when optimized CuAAC conditions that require 1 mM copper(I) are employed. Thus, as presently formulated, CuAAC is of limited use for labeling biomolecules in living systems. Cyclooctyne, the smallest stable cycloalkyne, reacted “like an explosion” when combined with phenylazide and enabled the detection of azides in living systems through strain-promoted [3+2] cycloaddition (Agard et al., 2004). Moreover, with the aim of improving the kinetics of the process, a series of compounds bearing electron-withdrawing fluorine atoms at the propargylic positions were investigated.

#### Competitive-ABPP

The non-specific binding is one of the main limitations of ABPP strategies. The photoreactive or electrophilic probes, even probes with higher concentration would in all probability label proteins non-specifically to some extent (i.e., not targets of the parent compound) (Wright and Sieber, 2016). To overcome this problem, the competitive strategy is receiving increasing attention. In competitive ABPP (Leung et al., 2003), a proteome is pre-incubated with parent compounds and subsequently with the activity-based probes, thus decreasing the binding of the probe with the target proteins by competing for the common binding site. The parent compounds are the prototype compounds before transforming to the probes, for example, Liao and his colleagues used SA to compete with the SA-probe to decrease its binding with IMPDH2 which demonstrated that they can interact with the same target (Liao et al., 2017). By this method, non-specific binding can be excluded, and only those sites that interact with the active site of the compound are analyzed. Some review papers have discussed its application and advantages and disadvantages (Willems et al., 2014; Wright and Sieber, 2016). With the development of advanced strategies, it has been applied in these strategies such as isoTOP-ABPP, fluoPol-ABPP and qNIRF-ABPP strategies, so we will discuss its application with these advanced strategies together in the next section.

### Advanced Strategies

#### isoTOP-ABPP

To identify the specific reactive amino acid sites of the target protein by using small molecules, Cravatt and co-workers developed a strategy called isoTOP-ABPP (isotopic tandem orthogonal proteolysis–ABPP) (Weerapana et al., 2010). This method uses isotope-labeled probes to achieve more reliable results compared to other quantitative protein profiling methods. This platform can simultaneously identify probe-labeled proteins and the exact sites of probe modification. Cysteine is the most intrinsically nucleophilic amino acid in proteins, and the activity of the protein is regulated by the modification of cysteine by endogenous and exogenous electrophiles. Iodoacetamide is a reagent classically used to react with cysteine and is often seen in proteomics; so, the Cravatt group used iodoacetamide to design a probe (Backus et al., 2016). The IA probe has an alkyne handle for “click chemistry” conjugation of probe-labeled proteins and isotopically labeled cleavable tags for quantitative mass spectrometry. Using this probe, researchers can quantitatively describe and profile the intrinsic reactivity of cysteine residues in native biological systems. Recently, Weerapana and his colleagues improved this IA probe. These researchers developed a pair of isotopically labeled iodoacetamide-alkyne probes, namely, IA-light and IA-heavy. These probes can be utilized for quantitative analysis of proteome samples and are easy to synthesize, especially compared to the isotopically tagged cleavable linkers (Abo et al., 2017). The iodoacetamide (IA)-based chemical probe has been used to concurrently quantify reactivity changes in hundreds of cysteines within cell lysates. However, the cytotoxicity of the IA group precludes efficient live-cell labeling, which is important for preserving transient cysteine modifications. To overcome this limitation, Weerapana and his colleagues developed a caged bromomethyl ketone (BK) electrophile, which shows minimal cytotoxicity and provides spatial and temporal control of electrophile activation through irradiation. Using this probe, these researchers were the first to describe reactivity changes associated with diverse cysteine modifications in living cells (Abo and Weerapana, 2015).

A competitive isoTOP-ABPP platform expands the application of this strategy for functional cysteines in proteomes. This platform has been used to identify the protein targets of HNE, 15d-PGJ2, and 2-HD and elucidate the cellular functions and mechanisms of action of these compounds (Wang et al., 2014). Fragment-based covalent ligand discovery coupled with competitive isoTOP-ABPP can rapidly lead to the discovery of lead small molecules and the identification of druggable sites. Using this platform, the Nomura group discovered some anti-cancer fragments and revealed the mechanisms of action of these fragments (Anderson et al., 2017; Bateman et al., 2017; Roberts et al., 2017). For example, this group confirmed one compound, DKM 2-93, which impairs pancreatic cancer cell survival and *in vivo* tumor growth, from a fragment-based cysteine-reactive ligand library and identified UBA5 as the target of this compound by covalently modifying the catalytic cysteine, thereby inhibiting the activity of the protein as an activator of the ubiquitin-like protein UFM1 to UFMylate proteins (Roberts et al., 2017).

Recent studies have shown that reactive scaffolds targeting other amino acids such as serine (Bachovchin and Cravatt, 2012), and lysine (Anderson et al., 2017; Hacker et al., 2017), can also be explored by using these platforms to discover unique and novel druggable sites in proteins. Anderson and coworkers developed a screening platform for lysine reactive fragments, which are dichlorotriazine-based covalent ligands, and screened this library to reveal small molecules that impair 231 MFP cancer cell survivals. Using this platform, they identified KEA1-97 and specific targets of KEA1-97 in 231 MFP proteomes and identified that this compound targets lysine 72 of thioredoxin, which disrupts the interaction of thioredoxin with caspase 3, activates caspases, and induces apoptosis.

#### FluoPol-ABPP

Target-based high-throughput screening (HTS) is essential for the discovery of small-molecule modulators of proteins. Typical screening methods rely on extensively tailored substrate assays for enzyme inhibitors or screens that profile cellular phenotypes. However, for those enzymes whose biochemical activity is not well characterized, such assays are not available. Competitive ABPP studies use SDS-PAGE as readout, limiting the applicability of such studies in HTS. Therefore, Cravatt and colleagues have developed a high-throughput competitive screening platform, namely, the fluopol-ABPP HTS assay, which can be used to select specific enzyme inhibitors, especially for enzymes with poorly characterized substrate or biological functions. The platform also combines high-throughput screening with identification of modes of action (Bachovchin et al., 2009). This strategy, based on a probe tagged with a fluorophore, combines fluorescent probes with competitive inhibition strategies. When the fluorescent probes react with target proteins, the fluorophore signal is strong and consistent; in the presence of a competitor, the probe is released and the signal is decreased. These results can be easily and rapidly measured; therefore, this assay is suitable for HTS. Fluopol-ABPP is a substrate-free approach that is ideally suited for studying enzymes for which no substrates are known.

Using this platform identified specific inhibitors of the substrate-free enzyme RBBP9 and the mechanistically distinct enzyme GSTO1 from a library of small-molecules (Bachovchin et al., 2009). Bachovchin et al. (2009) used the serine hydrolase-directed activity-based probe fluorophosphonate (FP)-rhodamine as the readout probe to select for specific inhibitors to purified RBBP9 from a library of 18,974 small molecules. From this screen, they identified 35 primary hits, and 20 compounds were confirmed via secondary gel-based screens. Finally, they identified emetine as a reversible RBBP9 inhibitor. This fluorophosphonate (FP)-rhodamine probe has also been used to explore other serine hydrolases, such as prolyl endopeptidase-like (PREPL) (Lone et al., 2011), phosphatase methylesterase-1 (PME-1) (Bachovchin et al., 2011a,b), and retinoblastoma-binding protein 9 (RBBP9) (Bachovchin et al., 2010).

Some other probes based on specific enzymes have also been used with the HTS-fluoPol-ABPP strategy. Bryan and his colleagues used a PAD-specific probe, namely, rhodamine-conjugated F-amidine (RFA), to develop an HTS assay. Using these assay conditions, they screened 2,000 compounds (5 μM final concentration) from an NIH validation set at The Scripps Research Institute in La Jolla, CA, United States (Pubchem AID 463073). Finally, they identified streptonigrin as an irreversible PAD4 inactivator (Knuckley et al., 2010). Tsuboi and his colleagues also combined their specific probe, a rhodamine-conjugated phenyl sulfonate ester (SE-Rh), with GSTO1 to identify GSTO1 inhibitors from a 300K+ compound library, and they confirmed an agent, KT53, that inactivates GSTO1 with excellent *in vitro* (IC50 = 21 nM) and *in situ* (IC50 = 35 nM) potency (Tsuboi et al., 2011).

#### qNIRF-ABPP

qNIRF-ABPP means quenched near-infrared fluorescent ABPP. Imaging agents that enable direct visualization and quantification *in vivo* have great potential value for monitoring chemotherapeutic responses and for early diagnosis and disease monitoring (Edgington et al., 2009; Garland et al., 2016). Fluorescent tags are heavily used in ABPP; however, the main limitation of these tags is the general fluorescence observed both during interaction with enzyme targets and when free in solution. To overcome this limitation, Matthew Bogyo’s group engineered probes with a highly efficient quenching group to inhibit the fluorophore group and make the probe intrinsically “dark”; such a probe emits a fluorescent signal only after covalently modifying a specific protease target, resulting in the loss of the quenching group (Blum et al., 2005). Finally, they synthesized the quenched probe GB117, which was attached the large but potentially cell-permeable quenching group QSY7 through a linker to improve the stability and potency of the probe. From fluorescent-imaging studies, they found that GB117 was mainly accumulated in lysosomes. GB117 probes are considered to be tools for cell-based imaging of cysteine cathepsin activity. However, the application of these probes for imaging in animals is limited. Therefore, these researchers combined their method with non-invasive imaging technology and generated a series of near-infrared fluorescent activity-based probes (NIRF-ABPs), which are better suited for *in vivo* imaging and target identification (Blum et al., 2007). These NIRF-ABPs contain Cy5 (646/664 nm excitation/emission), which is better suited for *in vivo* imaging owing to lower background fluorescence, and are insensitive to serum. The researchers synthesized the quenched probe GB137 and unquenched probe GB123 based on GB117 and GBB111 for application in *in vivo* imaging studies. An *in vivo* analysis of the quenched and unquenched probes was conducted to quantify the overall signal-to-background ratios for each probe in multiple animals; the results indicated that GB123 and GB137 generated similar overall signal-to-background ratios. However, some limitations still exist, such as the quenched probe achieved its maximum signal much more rapidly than the unquenched probe. Cathepsin protease activity is highly elevated in macrophages of vulnerable plaques and contributes to plaque instability. The researchers also explored the distribution of cathepsin in an atherosclerosis mouse model by using GB137 and GB123 (Abd-Elrahman et al., 2016). They compared these two probes by *in vivo* imaging and found that both probes showed distinct signals in the macrophage-rich ligated carotids; however, GB123 was also detected in the lymph nodes, aortic arch and heart and exhibited slower signal accumulation than GB137. These cathepsin ABPs represent a rapid diagnostic tool for macrophage detection in atherosclerotic plaque. An improved quenched fluorescent probe containing a phenoxymethyl ketone (PMK) electrophile with greater reactivity and broader selectivity compared to previously reported AOMK-based probes has been synthesized by Matthew Bogyo’s group (Verdoes et al., 2013).

## Discussion

Drugs that form covalent attachments with their targets have traditionally been considered to be conceptually distinct from conventional non-covalent drugs because the potential off-target reactivity could lead to undesirable side effects. However, covalent drugs have raised various concerns in the field of drug development (Singh et al., 2011; Bauer, 2015; Pichler et al., 2016). ABPP, a very powerful technique in target identification, has generated interest in covalent drugs and allows a more thorough investigation of the modes of action of individual drugs. ABPP is based on the activities of small molecules with a reactive group for binding and covalently modifying the active site of a certain enzyme class. Many ABPP probes have, so far, utilized electrophiles, including fluorophosphonates, sulfonates and epoxides, which exhibit preferences for nucleophilic groups in the active sites of several distinct enzyme classes (Bottcher and Sieber, 2008).

Now, ABPP has been thought as an enormous approach to explore drug targets, with the advanced strategies application, its application expand from drug targets identification to drug discovery. However, it stills exist some limitations, probes labeling non-specific proteins, which is the main issue in this field. Competitive ABPP strategy was commonly used to address this problem by comparison with control. With the quantitative proteomics application, the quantitative data can be used to cut off these background signals, and in general proteins are identified as hits by their enrichment in probe-treated sample over control groups. The other issue is the probe itself, probe-specific hits, which was difficult to deal with. Enrichment in the presence and absence of a competitor (typically the parent NP) is one approach widely used to test whether a protein is a probe-specific hit. Further work in this area may be helpful in providing resources to aid researchers in assessing whether putative targets are genuine or related to the probe moiety itself. To address this issue, follow-up validation of putative targets is very important.

isoTOP-ABPP can enable quantitative analysis of native amino acid reactivity and record changes in enzyme activity directly in native biological systems. It provides information about the post-translational modification of proteins and overcome the deficiency of conventional proteomic or genomic methods, which mainly focus on the expression level. Especially, a fragment-based ligand screening with competitive isoTOP-ABPP platform couples the identification of covalent ligands with the discovery of druggable hotspots. A reactivity-based chemical probe to map reactive, functional, and ligandable hotspots in complex proteomes is firstly needed such as iodoacetamide (IA) probe to label cysteine residues (Weerapana et al., 2010), fluorophosphonate (FP) probe for serine (Liu et al., 1999), sulfotetrafluoropheny (STP) for lysine (Hacker et al., 2017). An isotopically labeled valine for quantitative mass spectrometry (MS) measurements of labeled peptides across multiple proteomes is also important. Probe labeling efficiency is need consideration, for example, FP probes can react with >80% of mammalian metabolic serine hydrolases (Bachovchin and Cravatt, 2012).

FluoPol ABPP is a broadly applicable HTS platform for inhibitor discovery where the ability of compounds to block fluorescent activity-based probe labeling of proteins is monitored by fluorescence polarization and can be readily adapted for use with different classes of enzymes and ABPP probes. However, there are some important issues to be considered. A cognate activity-based probe has been developed before this platform. In addition, fluoPol-ABPP requires a substantial amount of purified protein, which may prove challenging for certain enzymes (e.g., transmembrane enzymes). Regardless, in cases where protein quantity is not limiting, fluoPol-ABPP is quite cheap, since the quantity of probe used per assay is negligible. A library of small molecules is another issue. This platform makes the ABPP technology useful not only for mechanism identification but also for compound discovery and will help us understand more about some poorly characterized enzymes and the inhibitors or activators of these enzymes.

It is important to visualize these diseased cells to enable diagnosis, facilitate surgical resection and monitor therapeutic response. Therefore, there is great opportunity to develop non-invasive imaging technologies for interventional surgical imaging and for diagnostic and therapeutic applications. The qNIRF-ABPP strategy provides a method for *in vivo* imaging. qNIRF-ABPs are potentially valuable novel imaging agents for disease diagnosis and are powerful tools for preclinical and clinical testing of small-molecule therapeutic agents *in vivo*, for the identification of specific therapeutic targets and biomarkers, and for monitoring the efficacy of small-molecule inhibitors (Joyce et al., 2004; Rosenthal et al., 2015; Garland et al., 2016).

## Conclusion

Activity-based protein profiling can provide an unbiased, global and quantitative analysis of protein binding partners. It has been used with different samples, including cell lysates, live cells, animal lysates, and even live animals. All these applications help us understand the interactions between compounds and organisms. With the applications of advanced strategies, ABPP has expanded its area from drug targets identification to drug discovery. The advanced strategies of ABPP open a new door for us, from target-based high-throughput screening to take images *in viv*o. isoTOP-ABPP strategy can provide us the global analysis of cysteine, serine and lysine reactivity even in living cells, which is important for preserving transient amino acids modifications. Fluopol-ABPP HTS assay overcome the traditional screening methods disadvantages relying on substrate assay and cellular phenotypes. It can be used for some poorly characterized enzymes to explore their inhibitors or activators. qNIRF-ABPP provides a method for *in vivo* imaging and is helpful for diagnosis, surgical resection and therapeutic response. The wide applicability of the above methods will provide more possibility to success for novel drug development, and expand more technical innovation in ABPP field. Finally, with advances in technology and through continuous improvement, chemical proteomic technology will remain at the forefront of drug discovery and target recognition.

## Author Contributions

SW wrote and edited this paper. YT and MW (third author) gave some convincing advice. MW (fourth author) checked and edited this paper. G-bS and X-bS designed this review.

## Conflict of Interest Statement

The authors declare that the research was conducted in the absence of any commercial or financial relationships that could be construed as a potential conflict of interest.
